# Detecting Intra-Field Variation in Rice Yield With Unmanned Aerial Vehicle Imagery and Deep Learning

**DOI:** 10.3389/fpls.2022.716506

**Published:** 2022-03-23

**Authors:** Emily S. Bellis, Ahmed A. Hashem, Jason L. Causey, Benjamin R. K. Runkle, Beatriz Moreno-García, Brayden W. Burns, V. Steven Green, Timothy N. Burcham, Michele L. Reba, Xiuzhen Huang

**Affiliations:** ^1^Department of Computer Science, Arkansas State University, Jonesboro, AR, United States; ^2^Center for No-Boundary Thinking, Arkansas State University, Jonesboro, AR, United States; ^3^University of Arkansas System Division of Agriculture, Little Rock, AR, United States; ^4^College of Agriculture, Arkansas State University, Jonesboro, AR, United States; ^5^U. S. Department of Agriculture, Department of Biological and Agricultural Engineering, University of Arkansas, Fayetteville, AR, United States; ^6^USDA Agricultural Research Service Delta Water Management Research Unit, Jonesboro, AR, United States

**Keywords:** convolutional autoencoder, remote sensing, UAS—unmanned aerial system, grain crop, precision agriculture

## Abstract

Unmanned aerial vehicles (UAVs) equipped with multispectral sensors offer high spatial and temporal resolution imagery for monitoring crop stress at early stages of development. Analysis of UAV-derived data with advanced machine learning models could improve real-time management in agricultural systems, but guidance for this integration is currently limited. Here we compare two deep learning-based strategies for early warning detection of crop stress, using multitemporal imagery throughout the growing season to predict field-scale yield in irrigated rice in eastern Arkansas. Both deep learning strategies showed improvements upon traditional statistical learning approaches including linear regression and gradient boosted decision trees. First, we explicitly accounted for variation across developmental stages using a 3D convolutional neural network (CNN) architecture that captures both spatial and temporal dimensions of UAV images from multiple time points throughout one growing season. 3D-CNNs achieved low prediction error on the test set, with a Root Mean Squared Error (RMSE) of 8.8% of the mean yield. For the second strategy, a 2D-CNN, we considered only spatial relationships among pixels for image features acquired during a single flyover. 2D-CNNs trained on images from a single day were most accurate when images were taken during booting stage or later, with RMSE ranging from 7.4 to 8.2% of the mean yield. A primary benefit of convolutional autoencoder-like models (based on analyses of prediction maps and feature importance) is the spatial denoising effect that corrects yield predictions for individual pixels based on the values of vegetation index and thermal features for nearby pixels. Our results highlight the promise of convolutional autoencoders for UAV-based yield prediction in rice.

## Introduction

Rice (*Oryza sativa*) is one of the most important staple food crops globally (Khush, 2001). However, efficient production remains a major challenge, and there is a growing need to increase yield gains per unit land area while conserving natural resources to meet current and future demands (Grassini et al., 2013). For example, nitrogen fertilization is one of the most challenging aspects of rice production, with recommended rates and timing depending significantly on cultivar, soil type, and other factors (Hardke, 2018). To optimize production while minimizing inputs and environmental impacts, real-time monitoring could enable more efficient identification of crop stress, yield projection, and decision-making throughout the season.

Remotely sensed images acquired by Unmanned Aerial Vehicles (UAVs) provide a flexible means to monitor crop stress and other production factors throughout the growing season. UAVs equipped with thermal sensors are sensitive to longwave infrared radiation (7,000–12,000 nm) and since transpiration rates and evaporative cooling decrease under water-limited conditions, thermal sensors are particularly suitable for early detection of drought stress (Maes and Steppe, 2019; Burns et al., 2022). UAVs can also be equipped with multispectral sensors that capture multiple spectral regions in relatively broad bands. In addition to red, green, and blue (RGB) bands, multispectral sensors capture wavelengths in the near-infrared (NIR) range (730–900 nm). A healthy vegetative canopy typically has very high reflectance in the NIR spectrum. Thus, multispectral imagery is particularly adept at assessing nutrient status for yield prediction (Maes and Steppe, 2019). Recently, state-of-the-art deep learning approaches are proving to be highly useful for yield prediction using analysis of images acquired by UAVs (Nevavuori et al., 2019), outperforming other methods.

An important consideration for the design of deep learning models from UAV-derived data is how to account for temporal variations in the spectral signatures of a developing crop. Rice canopy structure changes rapidly during vegetative growth, with early-season images mostly comprised of bare soil during seed germination, emergence, and seedling development. Approximately thirty days after planting at about the five-leaf stage, the first rice tiller appears (Hardke, 2018). At this time, flooding is initiated in the delayed-flood system used in Arkansas. Increased tillering coincides with an increase in green biomass, when the normalized difference vegetation index (NDVI), calculated based on reflectance in NIR and red bands (Table 1), begins to increase rapidly (Wang et al., 2014). Panicle initiation marks the beginning of reproduction. The developing panicle eventually emerges from the stem and is fully visible at heading when flowering begins. After pollination, the panicle develops, and the rice kernels fill, changing in color from light green to yellow and, ultimately tan, as the grains ripen and leaves senesce. Thus, spectral signatures steadily change with the development and maturation of the rice crop.

**TABLE 1:** Vegetation indices.

Index	Abbreviation	Equation^a^	References
Normalized difference vegetation index	NDVI	$\frac{\left(\rho_{NIR}-\rho_{r}\right)}{\left(\rho_{NIR}+\rho_{r}\right)}$	Tucker, 1979; Hatfield and Prueger, 2010; Sharma et al., 2015
Chlorophyll index green	CI_green_	$\frac{\left(\rho_{NIR}\right)}{\rho_{g}}-1$	Gitelson et al., 2005, 2003; Hatfield and Prueger, 2010
Red-edge normalized difference vegetation index	RENDVI	$\frac{\left(\rho_{NIR}-\rho_{RE}\right)}{\left(\rho_{NIR}+\rho_{RE}\right)}$	Gitelson and Merzlyak, 1997; Hatfield and Prueger, 2010
Green normalized difference vegetation index	GNDVI	$\frac{\left(\rho_{NIR}-\rho_{g}\right)}{\left(\rho_{NIR}+\rho_{g}\right)}$	Gitelson and Merzlyak, 1997; Hatfield and Prueger, 2010
Normalized area vegetation index	NAVI	$1-\frac{\left(\rho_{r}\right)}{\rho_{NIR}}$	Carmona et al., 2015
Triangle greenness index	TGI	−0.5[(670−480)*(ρ_*r*_−ρ_*g*_)	Hunt et al., 2011, 2013
		−(670−550)(ρ_*r*_−ρ_*b*_)]	

*^a^Reflectance (ρ) is measured at the wavelength denoted by the subscript: red (r), green (g), blue (b), red-edge (RE), and near-infrared (NIR).*

One strategy to account for variation in spectral and thermal indices across development is to let the model learn important features (such as changes in NDVI associated with developmental stage) during training. For example, Nevavuori et al. (2019) used Convolutional Neural Networks (CNNs) on wheat and malting barley fields to predict crop yield from derived vegetation indices and raw RGB data acquired from UAVs (∼0.3 m resolution). These CNNs were trained on data combined from nine fields, split into “early” and “late” growing season datasets based on the image collection date. Mean absolute percentage error was lower for models trained on early season (8.8%) compared to late season data (11.4%). These results suggest that relatively high performance can be achieved for yield prediction at the intra-field scale, even without more fine-grained consideration of plant developmental stage.

An alternative approach explicitly accounts for temporal aspects of variation in plant development in the model architecture. Recurrent neural networks (RNNs) are well-suited for sequential data due to the use of hidden states to capture relevant information from prior states. RNNs have been particularly successful for classification of land cover data from satellite imagery, due to the ability to leverage temporal patterns across image time series (Minh et al., 2018; Sun et al., 2019). Temporal data structures can also be considered with CNNs, when convolutions occur across images in the temporal dimension as well as in the spatial dimensions, and are called 3D-CNNs or temporal CNNs. Temporal CNNs demonstrated slightly improved performance compared to RNNs for land cover classification when considering spectral and temporal dimensions of the data only (Pelletier et al., 2019) and also when temporal, spectral, and spatial dimensions were considered (Li et al., 2017; Ji et al., 2018). While their utility is well-demonstrated for the task of land cover classification from satellite imagery, it is unknown whether temporal network architectures could also demonstrate improved accuracies for tasks such as intra-field prediction of crop yield based on higher pixel count images (as compared to satellite images) from UAVs.

**FIGURE 1:**
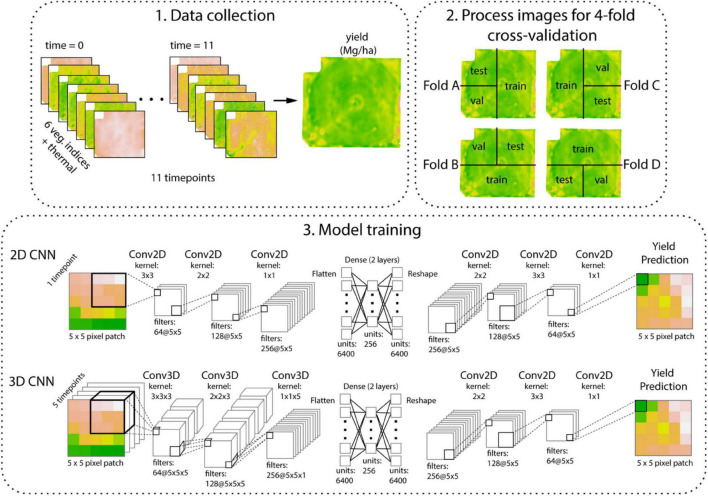
Study design. UAV images were collected at 11 time points during the season. Predictions were based on thermal data and six vegetation indices derived from red, green, blue, red-edge, and near-infrared bands (CIgreen, GNDVI, NAVI, NDVI, RENDVI, and TGI). The field was divided into training, test, and validation images as shown for four-fold cross-validation. These larger regions were divided into 5 × 5 pixel patches (50 cm resolution pixels). Convolutional neural networks (CNNs) used an autoencoder-like structure to predict yield on an output 5 × 5 pixel patch based on 5 × 5 pixel input images, each with seven features. Model training for the 2D-CNN was based on a single time point; 3D-CNN used the five time points centered around the reproductive phase of crop growth.

In this study, we assume that spatial variation in nutrient and water availability drives intra-field variation in spectral indices, and predict this variation will manifest as deviations from average conditions, observable from UAV imagery. We hypothesize that a model architecture that accounts for complex spatio-temporal patterns (e.g., 3D-CNN architecture) will be more informative for predicting intra-field yield variation compared to a spatial-only model (e.g., 2D-CNN architecture, Figure 1). We further determine whether deviations from average conditions matter most at certain time points, or if images taken during particular developmental stages are equally predictive of future yield. Finally, we characterize the nature of the benefit of the tested deep learning architectures for our dataset.

## Materials and Methods

### Study Site

Our study focuses on a single study site located in the state of Arkansas, which contributes approximately half of the agricultural land area harvested for rice grown in the United States (United States Department of Agriculture Economic Research Service [USDA], 2021). The study site is a 16-ha, zero-grade (0% slope) field within a large farm operation in Lonoke County (e.g., Runkle et al., 2019). The farm produces rice using a rice-after-rice (i.e., continuous rice) production system and a drill-seeded, delayed flood program and burns rice straw after harvest. Field soil is classified as silt loam: 33% Calhoun silt loam (Fine-silty, mixed, active, thermic Typic Glossaqualfs) and 66% Calloway silt loam (Fine-silty, mixed, active, thermic Aquic Fraglossudalfs) (United States Department of Agriculture Natural Resources Conservation Service, 2020).

### Rice Agronomics

The rice hybrid Gemini 214CL (Rice Tec, Inc., Alvin, TX) was drill seeded on 16 May 2019 using a seeding rate of 25 kg ha^–1^. Fertilizer was applied on 03 June (20 kg ha^–1^N and 52 kg ha^–1^ P_2_O_5_ using diammonium phosphate), 11 June (101 kg ha^–1^ K_2_O using potassium chloride), 12 June (101 kg ha^–1^ N using urea), and 25 June (50 kg ha^–1^ N using urea). To conserve water resources, the field was irrigated using alternate wetting and drying flood management (Bouman and Tuong, 2001; Henry et al., 2017).

The field was mechanically harvested on 14 September 2019 using a commercial combine and a circular harvest pattern with an 8.5-m header width. Rough rice yield was measured using a calibrated yield monitor (GPS-enabled John Deere Greenstar 3 2630 harvest monitor). Yield data were excluded from a 10-m buffer surrounding the field perimeter and associated drainage ditch. The data were checked and filtered using Yield Editor software (Sudduth and Drummond, 2007), and the harvest grain moisture content was 14.6%. ArcGIS software was used to develop a raster layer with a spatial resolution of 50 cm, using the spherical model, within the Kriging/CoKriging tool (Burrough, 2001). To further characterize the study site, throughout the growing season, growth, floodwater depth, canopy height and Leaf Area Index (LAI) were measured within 4 days of each flyover date (Table 2). Canopy height and floodwater depth were measured in five locations, while LAI was measured in two flagged locations with a total area of 1 m^2^ for each location using the LAI-2200C (LI-COR Biosciences). These measurements were performed on the north side of the field within a 15-m radius of each other, and the northern field edge.

**TABLE 2:** Flyover dates for the 2019 season.

Date	Days after planting	Growth stage	Average canopy height (cm) (*n* = 5)	Leaf area index (LAI) (*n* = 2)	Floodwater depth (cm) (*n* = 5)
Apr. 04	NA	NA	NA	NA	NA
May 21	6	Pre-emergence	NA	NA	0
June 13	29	Vegetative —tillering	25	0.26–0.30	0
June 29	45	Vegetative – Tillering	55	2.42–5.43	9–15
July 11	57	Reproductive—R0—panicle initiation	73	6.79–7.17	0–5
Aug. 01	78	Reproductive**—**R2**—**booting	82	7.16–7.27	0–3
Aug. 13	90	Reproductive**—**R4**—**flowering anthesis	111	6.51–6.57	15–23
Aug. 21	98	Grain filling**—**maturation**—**hard stage	104	6.23–6.47	10–14
Aug. 28	105	Grain filling**—**maturation**—**hard stage	104	NA	0
Sep. 07	115	Grain filling**—**maturation**—**hard stage	104	6.50–6.83	0
Sep. 13	121	One day before harvest	NA	NA	0

*Floodwater depth and LAI are given as ranges for the minimum and maximum values. For flyover days that occurred between dates when LAI or floodwater depth were measured, values are from the nearest day (within ± 4 days). Measurements were performed in the north side of the field within a 15-m radius.*

For further evaluation of trained models, we also considered a separate 27-ha field within the same farm in the 2020 growing season. This field was water seeded (seeds broadcasted from an airplane over a flooded field) with CL XL745 rice hybrid cultivar (Rice Tec, Inc., Alvin, TX) on 02 April 2020 using a seeding rate of 32.5 kg ha^–1^. Fertilizer was applied on 01 June (22 kg ha^–1^ N and 57 kg ha^–1^ P_2_O_5_ using diammonium phosphate), 11 June (52 kg ha^–1^ N using urea), 18 June (52 kg ha^–1^ N using urea) and 25 June (52 kg ha^–1^ N using urea). The field was also irrigated using alternate wetting and drying flood management to conserve water resources, and the rice residue was also burnt. The field was harvested on 17 August 2020 using the same combine previously described, and the harvest moisture was 15.6%. Field soil is classified as silty clay: Perry Silty Clay (Very-fine, smectitic, thermic Chromic Epiaquerts) (United States Department of Agriculture Natural Resources Conservation Service, 2020).

### Unmanned Aerial Vehicle Data Collection

A UAV with an Altum sensor (multispectral and thermal) was used for image data collection. Data were collected at approximately 7-day intervals, weather permitting (Table 2). The Matrice 210 V-2 quadcopter (DJI, Shenzhen, Nanshan District, China) was used and equipped with an Altum sensor (MicaSense, Seattle, Washington) to collect blue (B, 475 nm), green (G, 560 nm), red (R, 668 nm), red edge (RE, 717 nm), near-infrared (NIR, 840 nm), and thermal (11,000 nm) data. Data collection occurred within 2 h of solar noon local time. Prior to each flight, radiometric calibration images were captured (MicaSense, Seattle, Washington). Flight design parameters were calculated using the MicaSense flight calculator, while the Atlas Flight application was used to deploy flight missions (MicaSense, Seattle, Washington). The flight altitude was 120 m above ground level (AGL), and horizontal velocity was 10 m s^–1^ with 75% front/side overlap. The Pix4D mapper software (Pix4D Inc., Prilly, Switzerland) was used to stitch the raw imagery, producing orthomosaics. The model builder tool within ArcMap 10.7.1 (ESRI, 2011, Redlands, California) was used to calculate six vegetation indices, including CI_green_, Normalized Area Vegetation Index (NAVI), NDVI, Red-Edge NDVI (RENDVI), Green NDVI (GNDVI), and Triangular Greenness Index (TGI) based on the equations in Table 1. The six derived vegetation indices and the thermal layer were used as the input features for model training.

For the 2020 growing season, UAV data were collected on 05 July 2020 for the 27-ha field only, during booting stage.

### Image Processing

After producing orthomosaics and generating vegetation indices, images were further processed in R ver. 4.0 (R Core Team, 2020). Images were downsampled from 5 to 50 cm resolution, using the “aggregate” function of the raster package, and then split into 5 × 5 pixel tiles. This enabled faster processing of image data and a match to the spatial resolution of yield data. Images were then stacked across time. After cropping out the edges of the field and removing tiles with missing values, tiles were partitioned for four-fold cross-validation. Tiles were split into training (∼50%), test (∼25%), and validation (∼25%) datasets, in the four-fold shown in Figure 1. This strategy was used so that some field regions were never seen during training, rather than randomly assigning images to the test set, which would inflate model performance. For the two deep learning models, the validation set is used during model training, where model weights are updated each epoch if performance on the validation set improves; the test set is held out for the final evaluation after training. For the statistical models (linear, null, and XGBoost models), the training and validation sets can be considered equivalently. Non-overlapping tiles of 5 × 5 pixels were output as .csv files and then converted into .npy arrays for faster reading in Python 3.

### Model Training

#### Evaluation Metrics

To evaluate each model, four statistical parameters were used to assess model performance: Root Mean Squared Error (RMSE), coefficient of determination (*R*^2^), mean absolute error (MAE), and mean bias error (MBE), calculated as follows:


$$\text{RMSE}={\left[\frac{1}{n}\sum_{i=1}^{n}{\left(Y_{i}-{\hat{Y}}_{i}\right)}^{2}\right]}^{0.5}$$

$$R^{2}=\frac{{\left[\sum_{i=1}^{n}\left(Y_{i}-{\bar{Y}}_{obs}\right)\left({\hat{Y}}_{i}-{\bar{Y}}_{exp}\right)\right]}^{2}}{\sum_{i=1}^{n}{\left(Y_{i}-{\bar{Y}}_{obs}\right)}^{2}\sum_{i=1}^{n}{\left({\hat{Y}}_{i}-{\bar{Y}}_{exp}\right)}^{2}}$$

$$\text{MAE}=\frac{1}{n}\sum_{i=1}^{n}\left|{\hat{Y}}_{i}-Y_{i}\right|$$

$$\text{MBE}=\frac{1}{n}\sum_{i=1}^{n}\left({\hat{Y}}_{i}-Y_{i}\right)$$

where *Y*_*i*_ is the observed yield for pixel *i*, ${\hat{Y}}_{i}$ is the predicted yield for pixel *i*, *n* is the total number of pixels in the dataset, ${\bar{Y}}_{obs}$ is the mean observed yield for all pixels, and ${\bar{Y}}_{exp}$ is the mean predicted yield for all pixels. MAE and RMSE measure the average magnitude of difference in the observed and predicted response, with RMSE placing greater penalty on large errors. MBE is also a measurement of the error between the predicted and observed response but takes into account the sign of the errors. However, MBE should be interpreted with caution as large errors may cancel each other out if they are in the opposite direction. *R*^2^ represents the proportion of variance in the dataset that is explained by the model.

#### Null Models

As a baseline for comparison, we considered the difference between each pixel and a constant layer assigned the value of the mean yield calculated from all pixels assigned to the training set. Evaluation metrics for the null model on the training set vary slightly across time points as a result of differences in the number of missing values on different days.

#### Linear Models

We fit linear models to predict yield using seven predictors (all six vegetation indices and thermal rasters) using the lm() function from R version 4.0 (R Core Team, 2020).

#### XGBoost

We trained gradient boosted decision trees using the R implementation of XGBoost (Chen and Guestrin, 2016; Chen et al., 2021). This model was designed to capture complex interactions among predictor values, but did not consider spatial or temporal dimensions of our data structure. We did not perform extensive parameter tuning for every individual model, but chose parameter values that gave similar performance on training sets as observed for 2D-CNN models on a subset of data. Specifically, we used default settings with the exception of a slower learning rate (eta = 0.2), a maximum tree depth of 2 (max_depth = 2) to capture only pairwise interactions among predictors, and 200 rounds of training (nrounds = 200). The same parameter values were used to train all XGBoost models.

#### 2D-Convolutional Neural Networks

We included “spatial models” (2D-CNNs) to determine whether considering information from nearby pixels improved yield prediction. We developed a 2D-CNN with an autoencoder-like structure. An autoencoder is a neural network trained to encode data into a compressed representation and then reconstruct the original data from the encoded representation (Figure 1). Here, we take advantage of this type of architecture to predict an output 5 × 5 pixel image of yield, based on an input 5 × 5 image patch for the same location acquired by a UAV. Each image was associated with seven input features, corresponding to values from the thermal sensor or for a different vegetation index (Figure 1). We did not train models using data for 13 June 2020 due to a malfunction in the thermal sensor.

Our 2D-CNN was implemented in Python 3.8 using Keras with a TensorFlow v2.2.0 backend (Abadi et al., 2015; Chollet, 2015). The final architecture involved one sub-network of three convolutional layers for encoding, followed by two fully connected layers, and a second sub-network of three convolutional layers for decoding. The parameter specifications for each layer are shown in Table 3. A “ReLU” activation function was used for each layer in the network besides the last layer, which used a linear activation function. We used the “adam” optimizer and quantified loss based on the mean squared error. CNNs were trained for 50 epochs, and weights for models with the best performance on the validation set were saved to evaluate performance on the test set. Preliminary models were trained for up to 200 epochs, but only minor improvements in model performance were observed with additional training.

**TABLE 3:** Parameters for CNNs.

Layer	Parameters (2D-CNN)	Parameters (3D-CNN)
Conv2D (for 2D-CNN) or Conv3D (for 3D-CNN)	Filters = 64	Filters = 64
	Kernel_size = (3,3)	Kernel_size = (3,3,3)
	Padding = “same”	Padding = “same”
	Activation = “relu”	Activation = “relu”
	Input_shape = (5,5,7)	Input_shape = (5,5,5,7)
Conv2D (for 2D-CNN) or Conv3D (for 3D-CNN)	Filters = 128	Filters = 128
	Kernel_size = (2,2)	Kernel_size = (2,2,3)
	Padding = “same”	Padding = “same”
	Activation = “relu”	Activation = “relu”
Conv2D (for 2D-CNN) or Conv3D (for 3D-CNN)	Filters = 256	Filters = 256
	Kernel_size = (1,1)	Kernel_size = (1,1,5)
	Padding = “valid”	Padding = “valid”
	Activation = “relu”	Activation = “relu”
Reshape	Target_shape = (5,5,256)	
Flatten	NA	
Dense	Units = 256	
	Activation = “relu”	
Dense	Units = 5,400	
Reshape	Target_shape = (5,5,256)	
Conv2D	Filters = 128	
	Kernel_size = (2,2)	
	Padding = “same”	
	Activation = “relu”	
Conv2D	Filters = 64	
	Kernel_size = (3,3)	
	Padding = “same”	
	Activation = “relu”	
Conv2D	Filters = 1	
	Kernel_size = (1,1)	
	Padding = “same”	
	Activation = “linear”	

*Each Keras layer refers to a building block of the neural network, including convolution layers (Conv2D and Conv3D), reshaping layers (Flatten and Reshape), and fully connected layers (Dense). Besides the last layer, all layers used a rectified linear unit (“relu”) activation function that directly outputs the input, if positive, or zero otherwise.*

#### 3D-Convolutional Neural Networks

**FIGURE 2:**
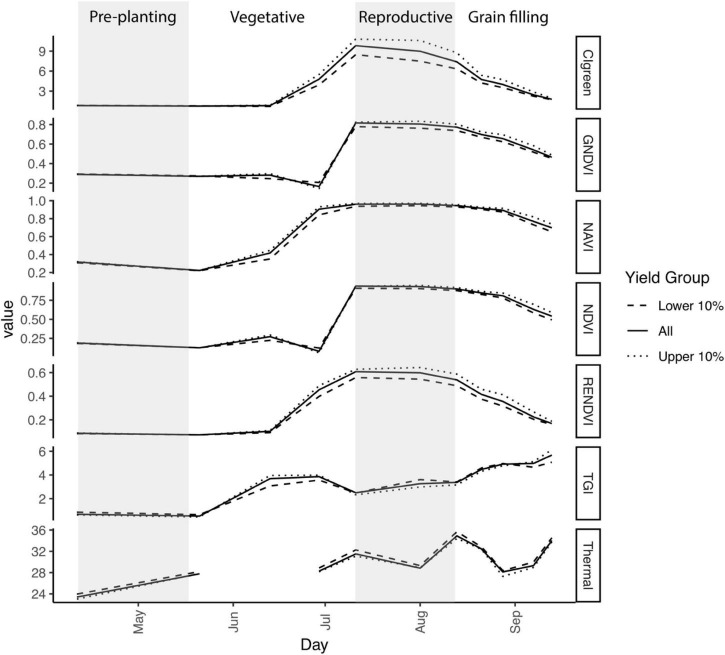
Variation in temperature and six vegetation indices over time in 2019. Gray shaded areas indicate time prior to seeding and the reproductive phase (panicle initiation through flowering anthesis). Additional details regarding growth stages for each flyover date are provided in Table 2. First flooding occurred on 29 June, resulting in a dip in NDVI and GNDVI. Plotted values are the mean for 50 cm × 50 cm pixels with yield in the lower 10% (*n* = 52,649), the upper 10% (*n* = 52,537), or all pixels (*n* = 526,735). Thermal was not available for the flyover on 13 June.

To determine whether considering information from nearby time points improved yield prediction, we developed “spatial-temporal models” (3D-CNNs). For this analysis, we used 5 days beginning just prior to the reproductive phase (flyover dates from 29 June 2019 through 21 August 2019), which ended approximately 3 weeks prior to harvest and also included the days that we anticipated to be most informative with respect to variation in vegetation indices (Figure 2). We also tested 3D-CNNs that included all 11 time points, but found early on during testing that they primarily learned to weight features from the final time point, just prior to harvest. Our 3D-CNNs were designed to have a parallel structure to our 2D-CNNs with the exception that convolutions occurred in three dimensions in the encoding stage of the network (Figure 1).

#### Computational Infrastructure

The XGBoost, linear, and null models were trained in minutes or less on a personal desktop computer (16 Gb RAM; Intel Core i5 3 GHz processor). Each 2D-CNN was trained on a single node of the Trestles cluster at the University of Arkansas High Performance Computing Center (AHPCC). Each of these nodes is equipped with 64 Gb of memory and four AMD 6136 2.4 GHz CPUs for a total of 32 cores; 2D-CNNs required approximately five hours to train (eight CPU hours) and a maximum of 10 Gb of virtual memory. We used the same computing infrastructure for training 3D-CNNs as for 2D-CNNs; each 3D-CNN required approximately 18 hours (24 CPU hours) to train.

### Model Comparison

In comparing our models, we sought to answer three questions: (1a) Do model architectures that capture spatial information improve yield prediction over traditional statistical learning approaches? (1b) If so, do models that also include data from multiple time points improve yield prediction over models that only include spatial information? (2) Which day(s) have the strongest signal for deep-learning based yield prediction? and (3) What are the most important spectral features for prediction?

For the first two questions, we compared average test set RMSE across time points for all models. To qualitatively determine the impact of different model architectures on yield predictions, we also projected models to field scale. Input images containing vegetation indices and thermal layers were processed in R as described for model training. CNN models were loaded into R using the “reticulate” package to enable interoperability between R and python codes (Ushey et al., 2020). Predictions for each tile were generated iteratively and tiled together for the prediction map for the field.

For the last question, we determined the relative importance of each feature for the trained 2D-CNNs by removing variation observed for that feature in input images from the test set. To “blank” variation in a feature, all actual values for that feature in each tile were replaced by the mean value observed across all pixels in the test set. Test set RMSE was then determined using the function call to Keras “evaluate” (Chollet, 2015).

### Code Availability

**FIGURE 3:**
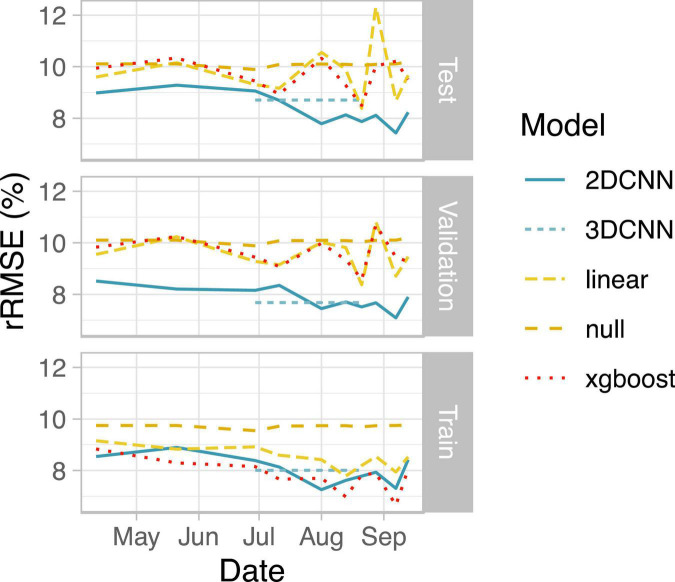
Summary of model performance. Root mean squared error (RMSE) relative to the mean yield for the field is shown as the average across all four data folds. Note that results for the 3DCNN are for a single model based on input from five time points together, with relative RMSE (rRMSE) shown as a constant value across the five included time points.

**FIGURE 4:**
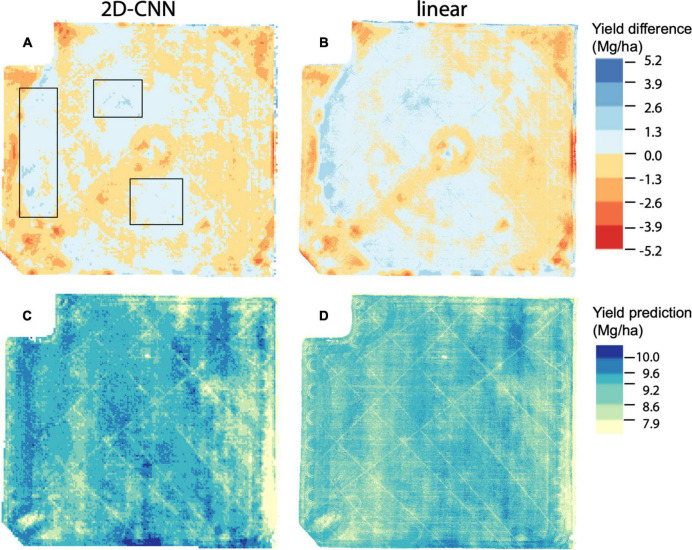
Yield prediction maps based on data for 01 Aug 2019, for models trained on the fold B dataset. Data for 2D-CNN is shown in **(A,C)** whereas data for linear model is shown in **(B,D)**. Prediction error (yield difference) is shown in **(A,B)** and represents observed yield minus predicted yield; **(C,D)** show predicted yield. Black boxes in **(A)** indicate regions described in main text where yield was underpredicted to a considerably lesser degree in the 2D-CNN model compared to the linear model. For **(A,B)**, breakpoints for color scale are evenly spaced. For **(C,D)**, breakpoints for the color scale are chosen based on the 10th, 25th, 50th, 75th, and 90th percentile values of observed yield. Mg/ha: megagrams/hectare.

**FIGURE 5:**
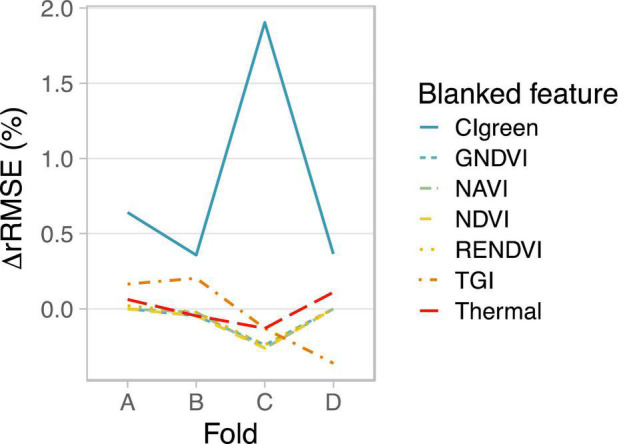
Feature importance for model training on images from 01 Aug 2019. To blank observed variation in a feature, actual values were replaced by the mean value across all pixels observed for that feature. ΔRMSE was calculated by subtracting the test set RMSE of the original model with no feature blanked from the blanked feature model. ΔRMSE is given relative to the mean yield (ΔrRMSE). Larger, more positive ΔrRMSE values indicate higher feature importance for the respective feature.

Python and R code used to process data, train and evaluate models, and recreate Figures 2–5, is available at https://github.com/em-bellis/XASU_rice.

## Results

### Rice Yield Variation

We first characterized intra-field variation in yield in the 2019 growing season. Rough rice yield was 9.06 ± 0.9 Mg ha^–1^ (mean ± std. dev.) measured across 526,735 grid cells at 50-cm resolution. As expected, vegetation indices varied with rice crop developmental stage and differed between high- and low-yielding areas of the field (Figure 2). CI_green_ and RENDVI showed the greatest contrast during reproduction, peaking at booting stage (CI_green_) or flowering (RENDVI) in the highest-yielding areas of the field. TGI values also differed among high- and low-yielding areas of the field, particularly during vegetative growth and booting stage (Figure 2).

### Spatial vs. Non-Spatial Models

We next evaluated the ability of deep learning-based, spatially explicit models to predict yield from vegetation index and thermal feature information. Compared to the null model, all models showed improved performance during training, indicating that vegetation indices and thermal features provided useful information for predicting yield (Figure 3). Linear models performed worst for the training set data for eight out of 10 days. Non-spatial (XGBoost) models performed best on training set data for six of 10 days, reaching the best performance on images acquired a week prior to harvest (Figure 3).

Performance on test sets, however, revealed a clear benefit of our deep learning-based spatial models for predicting grain yield both in terms of higher accuracy and lower variability in predictions across folds (Figure 3 and Table 4). Similar ranking of models was observed for all metrics (Table 4). 2D-CNNs trained on images taken during booting stage (01 Aug) or later showed the best performance (RMSE: 7.4–8.2% of mean yield; Figure 3). Average test RMSE of XGBoost models during these same developmental stages was higher, ranging from 8.5 to 10.3% of the mean yield. Performance of XGBoost models was also highly variable across folds, with standard deviation up to 7.4% of the mean yield vs. 4.2% in 2D-CNNs (*n* = 4 folds, based on observations over all time points). The difference in performance of XGBoost models on training and test sets may be indicative of overfitting. However, even for days on which performance of CNNs and XGBoost models on the training set was nearly identical (i.e., 29 June, 21 Aug, and 28 Aug), 2D- and 3D-CNNs showed markedly better performance on the test set (Figure 3). 2D-CNNs also outperformed other models with respect to MAE and *R*^2^, though not with respect to MBE, suggesting that although they were more accurate, 2D-CNNs tended to overpredict slightly more than other models (Table 4). Models trained on images collected from the booting stage or later performed best, though 2D-CNNs trained on images from earlier time points also performed notably better than other models (RMSE: 8.7–9.3% of mean yield; Figure 3).

**TABLE 4:** Evaluation of model performance on the test set for single-day models (trained on data at late booting stage) and 3D-CNN.

Model	Fold	RMSE (Mg/ha)	MBE (Mg/ha)	MAE (Mg/ha)	*R* ^2^
Null	A	0.93	−0.50	0.79	n.d.
	B	0.75	0.08	0.57	n.d.
	C	0.80	0.30	0.60	n.d.
	D	0.83	0.13	0.62	n.d.
	*Mean*	*0.83*	*0.00*	*0.65*	*n.d.*
Linear	A	1.06	−0.78	0.93	0.17
	B	0.70	0.07	0.51	0.18
	C	0.94	0.59	0.70	0.15
	D	0.83	0.10	0.63	0.04
	*Mean*	*0.88*	*−0.01*	*0.69*	*0.14*
XGBoost	A	0.88	−0.45	0.72	0.11
	B	0.74	−0.02	0.53	0.19
	C	0.83	0.50	0.61	0.22
	D	0.83	0.11	0.63	0.04
	*Mean*	*0.82*	*0.04*	*0.63*	*0.14*
2D-CNN	A	0.73	−0.19	0.57	0.18
	B	0.68	0.13	0.47	0.29
	C	0.63	0.02	0.45	0.30
	D	0.84	0.28	0.62	0.10
	*Mean*	0.72*	0.06*	*0.53*	*0.22*
3D-CNN	A	0.80	−0.37	0.67	0.08
	B	0.67	0.17	0.48	0.27
	C	0.77	−0.24	0.61	0.37
	D	0.90	0.45	0.67	0.06
	*Mean*	*0.79*	*0.00*	*0.61*	*0.20*

*Indicates significant difference in mean value between 2D-CNN and linear model only (p ≤ 0.05; one-way ANOVA).

*RMSE, root mean squared error; MBE, mean bias error; MAE, mean absolute error;R^2^, coefficient of determination. n.d., not defined (observed ∼ predicted yield is a vertical line for the null model).*

To further evaluate the benefit of our spatial models, we projected predictions from 2D-CNNs trained during the booting stage to field scale (Figure 4). This analysis suggested that a main benefit of the 2D-CNN model, compared to models that do not incorporate information from nearby pixels, may be a spatial denoising effect of the 2D-CNN. Compared to less complex models, CNNs were less likely to underpredict yield, particularly where yields were higher (Figures 4A,B).

### Spatial vs. Spatial-Temporal Models

We observed comparable performance for the two deep learning models using the tested architectures. Average test RMSE for 3D-CNNs only exceeded that of 2D-CNNs on 29 June and 11 July, likely due to the fact that the 3D-CNN model also included data from the more informative, later time points (Figure 3). Our results suggest that 2D-CNN models provide a benefit for the task of yield prediction in rice over simpler models and may offer similar performance to some deep learning architectures that incorporate data from multiple timepoints. Future studies may find further benefit of temporal network architectures relative to the 2D-CNNs tested here, for example by altering the intervals of the selected time points.

### Spectral Feature Importance

The cost of a UAV increases with the number of sensors it carries and sensor complexity. To assess if it is possible to achieve similar prediction accuracy with fewer sensors or bands, we determined the importance of each index on model performance of booting stage 2D-CNNs. Booting stage is early enough to be useful to the farmer, such as for determining the need for late boot nitrogen fertilization of rice hybrids (Hardke, 2018). It was also found to have one of the lowest RMSE values (Figure 3).

At booting stage, CI_green_ was the most important feature (index) for predicting rice yield with 2D-CNNs (Figure 5). Depending on the fold, test-set RMSE increased by 0.03–0.17 Mg ha^–1^ when variation among pixels in CI_green_ was removed, consistent with high variation in CI_green_ among yield groups at this time point (Figure 2). TGI and thermal information were also important, but only for some data folds (Figure 5). Other indices appeared to matter little to overall model performance, with negligible or even positive effects on model performance when observed variation in those features was removed (Figure 5). However, since calculation of TGI relies on three bands (red, green, blue), CI_green_ relies on two bands (green, NIR), and thermal information was also useful for some models (Figure 5), a UAV equipped with all sensors is recommended to achieve levels of performance reported here on other datasets.

### Generalization to New Datasets

**FIGURE 6:**
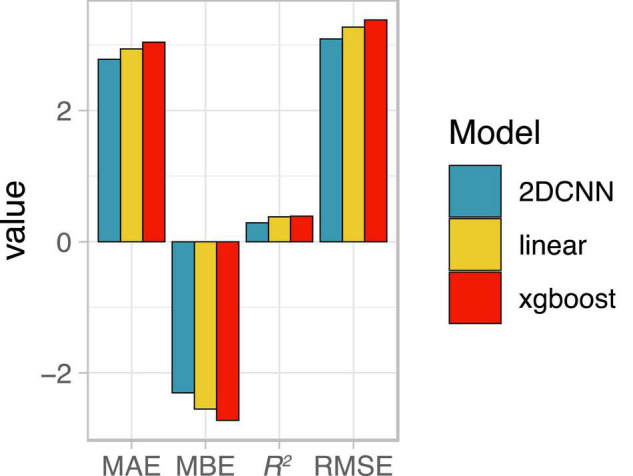
Average performance of late booting stage models from 2019, evaluated on a separate nearby field in the 2020 growing season. MAE, mean absolute error; MBE, mean bian error; *R*^2^, coefficient of determination; RMSE, root mean squared error; 2DCNN, two-dimensional convolutional neural network.

To explore the extent to which our findings may generalize to new contexts, we evaluated performance of late booting stage models from 2019 (Figure 4) on a separate, nearby field imaged in the 2020 growing season. All 2019 models underpredicted yield in 2020 (Figure 6), consistent with substantially higher mean yield for the 27-ha field compared to the training dataset (11.4 vs. 9.1 Mg/ha). Among all single-day UAV-based models, the 2D-CNN model had the highest accuracy, indicating it was also more translatable to a different field and growing season compared to the other models (Figure 6).

Further improving performance in new contexts will require a greater diversity of training images for different rice cultivars, growing seasons, soil types, and management conditions. To inform future experimental design, we determined the extent to which similar performance could be expected for models trained on smaller datasets. A subset of 1,000 tiles was randomly selected from the fold B dataset (Figure 1; ∼10% of tiles compared to full-scale training). After 50 epochs, RMSE as low as 0.77 Mg/ha was observed for the smaller training set; in contrast, lower RMSE (0.68 Mg/ha) was achieved within 50 epochs for the full training set. Given the modest increase in RMSE with the smaller dataset, it may be prudent to train future deep learning models using at least a similar-sized training set (∼7 ha) as the full-scale training set used here.

## Discussion

In this study, we present an autoencoder-like CNN architecture for intra-field prediction of rice yield. The best single-day model showed improved performance compared to simpler models trained on the same data, and comparable or improved performance to similar UAV-based studies in wheat and barley (Nevavuori et al., 2019), rice (Yang et al., 2019; Wan et al., 2020; Duan et al., 2021), and soybean (Maimaitijiang et al., 2020; Table 5). With respect to yield prediction in rice, we report slightly better performance of our late booting stage 2D-CNN compared to Yang et al. (2019), after accounting for higher average yield in our study [RMSE of 0.72 (Table 4) vs. 0.76 Mg/ha (Yang et al., 2019)]. With respect to RMSE, we report slightly lower performance for rice yield prediction compared to two other studies; however, these studies tested performance using leave-one-out cross-validation (Duan et al., 2021) or random samples distributed throughout the field (Wan et al., 2020), which could inflate performance compared to the spatially explicit strategy for cross-validation used here (Figure 1). Compared to other studies, *R*^2^ values for our model were relatively low, likely because of greater amount of overall yield variation in other studies due to experimental nitrogen treatment (Wan et al., 2020) and differing management practices (Yang et al., 2019). Our findings additionally suggest a benefit of autoencoder-like 2D-CNNs for spatial denoising of yield predictions by incorporating information from nearby pixels. With the exponential rise in adoption of UAVs for remote sensing in agriculture (Maes and Steppe, 2019), this study provides timely guidance for future large-scale training data collection efforts and their integration with development of deep-learning models.

**TABLE 5:** Model performance for comparable studies using UAV imagery for yield prediction.

References	Crop	Model	Performance	Description
This study	Rice	2D-CNN	7.9% (rRMSE)	Yield predicted from thermal and six VIs using data at late booting stage.
			5.8% (MAPE)	Performance based on 4-fold cross validation from the same field and season.
			0.22 (*R*^2^)	
Duan et al. (2021)	Rice	Neural network	5.3–7.1% (rRMSE)	Yield predicted on two individual VIs from 6 or more imaging days.
			0.48–0.62 (*R*^2^)	Performance based on leave-one-out cross validation from the same field and season.
Wan et al. (2020)	Rice	random forest	2.75% (rRMSE)	Yield predicted from four RGB- and multispectral-derived features. Data set included substantial yield variation due to experimental nitrogen treatment.
			0.83 (*R*^2^)	Performance based on random held-out set from the same field and season.
Yang et al. (2019)	Rice	2D-CNN	26.6% (MAPE)	Yield predicted from raw RGB and multispectral imagery at ripening stage.
			0.49 (*R*^2^)	Performance based on held-out set of independently managed plots from the same season.
Maimaitijiang et al. (2020)	Soybean	2D-CNN	15.9% (rRMSE)	Yield predicted from 72 features derived from multispectral, thermal, and RGB sensors on a single day. Data set included substantial yield variation due to cultivar-specific differences.
			0.72 (*R*^2^)	Performance based on held-out set from the same field and season.
Nevavuori et al. (2019)	Wheat/barley	2D-CNN	8.8–12.6% (MAPE)	Yield predicted from RGB or a single VI measured on a single day. UAV data were combined for two crops, nine fields, and multiple imaging dates. Images for “early” or “late” season models were sub-sampled, shuffled, and split into test and train sets.
				

*rRMSE, relative root mean squared error; MAPE, mean absolute percentage error.*

Surprisingly, we observed similar performance for yield prediction for 2D-CNNs as for a 3D-CNN architecture using data from multiple time points. However, 3D-CNN architectures may show a greater increase in performance if trained on diverse datasets that include multiple rice cultivars and environments, particularly if there are significant cultivar- or environment-specific differences in the pattern of vegetation index change over time (Duan et al., 2021). Exploring the benefit of 3D-CNNs for better generalization across climate zones and cultivars is a promising area for future work, since a primary benefit of these architectures may be the ability to take into account shifts in phenology across different climates and cultivars. The dataset utilized here, which focuses on fine-scale yield prediction across a large, heterogeneous field for a single year, minimizes variation due to cultivar and environmental differences, and so any temporal variation in vegetation indices associated with yield may not contribute to a strong spectral signature in the dataset. Our pre-processing pipeline also does not include any explicit classification of soil- or weed-derived pixels, or inclusion of canopy structure/texture features (e.g., Maimaitijiang et al., 2020), which could also impact the relationships among timepoints and the relative performance of 3D-CNNs. Use of vegetation index features that are less sensitive to saturation and soil background effects (e.g., Yang et al., 2019) is another strategy which might influence 3D-CNN performance relative to 2D-CNNs.

Our results also highlight the potential for UAVs to support management recommendations even during early growth stages (Nevavuori et al., 2019). Although the best single-day models were obtained during booting stage or later, the 2D-CNNs showed considerably better performance than other models even when trained on data acquired during vegetative growth stages (Figure 3). This difference in prediction for 2D-CNNs vs. other models was observable even prior to planting when the performance of single-day models was surprisingly competitive with models based on information from later in the season (Figure 3). Other studies demonstrate the success of 2D-CNNs for corn yield prediction based only on pre-season variables, including soil electroconductivity maps and satellite imagery acquired after soil tillage (Barbosa et al., 2020). Bare-soil images taken by UAVs prior to planting may also capture features that correlate with soil properties important to yield (Khanal et al., 2018).

For future large-scale efforts on a greater diversity of rice cultivars from different fields, regions, years, and management conditions, our results suggest it may be worthwhile to focus data collection at time points just prior to common crop management intervention points. In turn, growth-stage specific single-day models can be trained using these data. For example, Arkansas currently recommends nitrogen fertilization after internode elongation (for some cultivars) or at late booting (for hybrid cultivars) (Hardke, 2018); the most robust models might be explicitly trained for optimal performance at those stages. The use of growth-stage specific models may be particularly valuable because the importance of different vegetation indices for yield prediction varies over time (Figure 2; Duan et al., 2019). Compared to 3D-CNNs, 2D-CNNs would also require less computational and environmental resources for training (Strubell et al., 2019; Henderson et al., 2020; Bender et al., 2021) and fewer flyovers to generate yield predictions when models are deployed.

Further contributing to the simplicity of our deep learning models is the lower resolution of images used for the models in our study (50 cm) compared to the resolution of images available from the Altum sensor (5 cm). Using down-sampled images, our 2D-CNNs reached maximum performance relatively early during training. Contributing to this, the true relationship between yield and vegetation indices may be relatively simple; high linear correlations with yield are often reported (Duan et al., 2019). Furthermore, higher resolution of input images would not match the scale of accuracy of yield maps generated using data collected by commercial harvesters (Figure 1). Conversely, without a combine yield monitor, it would be very difficult to acquire a sufficient volume of labeled data needed to train deep learning models.

If the relationship between vegetation indices and yield is relatively simple, and the resolution of imagery used here precludes automated detection of individual objects in images, what is the utility of our CNN architectures for yield prediction? One of the primary benefits may be an image denoising effect of the autoencoder-like model architecture. Autoencoders have been widely successful for image denoising for a variety of applications (Xie et al., 2012). Robustness to partial destruction of the input is a characteristic of particular interest for denoising autoencoders (Vincent et al., 2008). Our study suggests that similar architectures are also useful for denoising “outputs.” For example, although yield maps used for training included noise (e.g., circular impressions due to the driving pattern of the combine harvester), these patterns are absent in prediction maps (Figures 4C,D). Future models trained to predict yield using higher resolution images from UAVs might benefit from a two-stage approach, where yield maps from a combine harvester first undergo error correction using the model architecture presented here (Figure 1).

Taken together, our study highlights the benefits of relatively simple CNN architectures for yield prediction in rice using remotely sensed images. Incorporating such models into data analysis pipelines could balance the overall costs of data collection and model training and demonstrates the potential benefits of deep learning for sustainable agriculture and precision management.

## Data Availability Statement

The original contributions presented in the study are included in the article/supplementary material, further inquiries can be directed to the corresponding author/s.

## Author Contributions

AH, EB, JC, BR, TB, MR, and XH designed the study. BR and MR directed the experimental study. BR directed collection of yield data and other study site agronomic information, with help from BM-G. AH directed collection and processing of UAV images, with help from BB. EB developed CNNs with input from XH and JC. The manuscript was drafted by EB with input from AH, BR, MR, VG, JC, and BM-G. All authors contributed to manuscript revisions and read and approved the final version.

## Conflict of Interest

The authors declare that the research was conducted in the absence of any commercial or financial relationships that could be construed as a potential conflict of interest.

## Publisher’s Note

All claims expressed in this article are solely those of the authors and do not necessarily represent those of their affiliated organizations, or those of the publisher, the editors and the reviewers. Any product that may be evaluated in this article, or claim that may be made by its manufacturer, is not guaranteed or endorsed by the publisher.
